# Automated Euler number of the alveolar capillary network based on deep learning segmentation with verification by stereological methods

**DOI:** 10.1111/jmi.13390

**Published:** 2025-01-31

**Authors:** Julia Schmidt, Jonas Labode, Christoph Wrede, Yannick Regin, Jaan Toelen, Christian Mühlfeld

**Affiliations:** ^1^ Hannover Medical School Institute of Functional and Applied Anatomy Hannover Germany; ^2^ Biomedical Research in Endstage and Obstructive Lung Disease Hannover (BREATH) German Center for Lung Research (DZL) Hannover Germany; ^3^ Hannover Medical School Research Core Unit Electron Microscopy Hannover Germany; ^4^ Department of Development and Regeneration KU Leuven Leuven Belgium

**Keywords:** 3D reconstruction, alveolar capillary network, deep learning, Euler number, image segmentation, serial block‐face scanning electron microscopy, stereology

## Abstract

Diseases like bronchopulmonary dysplasia (BPD) affect the development of the pulmonary vasculature, including the alveolar capillary network (ACN). Since pulmonary development is highly dependent on angiogenesis and microvascular maturation, ACN investigations are essential. Therefore, efficient methods are needed for quantitative comparative studies. Here, the suitability of deep learning (DL) for processing serial block‐face scanning electron microscopic (SBF‐SEM) data by generating ACN segmentations, 3D reconstructions and performing automated quantitative analyses based on them, was tested. Since previous studies revealed inefficient ACN segmentation as the limiting factor in the overall workflow, a 2D DL‐based approach was used with existing data, aiming at the reduction of necessary manual interaction. Automated quantitative analyses based on completed segmentations were performed subsequently. The results were compared to stereological estimations, assessing segmentation quality and result reliability. It was shown that the DL‐based approach was suitable for generating segmentations on SBF‐SEM data. This approach generated more complete initial ACN segmentations than an established method, despite the limited amount of available training data and the use of a 2D rather than a 3D approach. The quality of the completed ACN segmentations was assessed as sufficient. Furthermore, quantitative analyses delivered reliable results about the ACN architecture, automatically obtained contrary to manual stereological approaches. This study demonstrated that ACN segmentation is still the part of the overall workflow that requires improvement regarding the reduction of manual interaction to benefit from available automated software tools. Nevertheless, the results indicated that it could be advantageous taking further efforts to implement a 3D DL‐based segmentation approach. As the amount of analysed data was limited, this study was not conducted to obtain representative data about BPD‐induced ACN alterations, but to highlight next steps towards a fully automated segmentation and evaluation workflow, enabling larger sample sizes and representative studies.

## LAY DESCRIPTION

Bronchopulmonary dysplasia (BPD) is a developmental disruption of the lung, often found in premature newborns. BPD and other diseases affect the development of the blood vessels in the lung. This includes the alveolar capillary network (ACN), a mesh‐like structure of blood vessels that envelops the end segments of the airways. It facilitates the exchange of gas between air and blood. The development of the lung is highly dependent on the development of the ACN. Thus, efficient methods to analyse the ACN are needed to perform comparative studies between healthy and diseased lungs. The ACN can be imaged using scanning electron microscopy (SEM). This is a technique to take high‐resolution images of a sample. Individual images can then be combined to a 3‐dimensional (3D) image of the sample. From the 3D sample image, a 3D model of the ACN can be generated. Therefore, the ACN needs to be segmented on the sample images. Segmentation is a method to digitally label structures in images. It is a time‐consuming process when done manually or only with the help of basic image filters. Deep learning (DL) is another computer tool that can be used for segmentation tasks. It makes use of complex structures inspired by biological brains. To use DL methods, a training procedure using already segmented images is necessary. In this study, segmentation results of the ACN generated by a DL approach were compared to results generated by an established method using basic image filters. The segmentation results were used to generate 3D models of the ACN.

To understand what changes the ACN in diseases, it needs to be measured and compared to healthy ACNs. Measuring can be performed in the 2D SEM images using stereology. This is a set of manual methods for taking unbiased sample measurements of 3D structures. It ensures representative results. Given a 3D ACN model, measurements can also be taken automatically using a computer program. In this study, automatically obtained measurements were compared to stereological measurements to check if the automated method is able to obtain reliable results.

It was shown that the DL approach made the ACN segmentation process more efficient. Furthermore, the automatically obtained ACN measurements were reliable.

## 1 INTRODUCTION

The site of pulmonary gas exchange are the alveoli, where air and blood are separated only by a thin sheet of cellular processes and extracellular space. The interalveolar septa contain the alveolar capillary network (ACN), enveloping the alveoli.^1^, ^2^ In general, the ACN forms a vast network of blood vessels and is characterised by its complex 3D architecture and sheet‐like structure, consisting of interlinked short blood vessel segments, interrupted by connective tissue pillars.^1^, ^3^, ^4^, ^5^, ^6^, ^7^, ^8^

A variety of lung diseases impact the pulmonary vasculature, thus also affecting the ACN and consequently the process of gas exchange.^8^, ^9^, ^10^ One disease that is known to alter the pulmonary vasculature is bronchopulmonary dysplasia (BPD), one of the most common negative consequences of premature birth. This disease has the potential to result in serious short and long‐term complications.^11^, ^12^, ^13^ The vascular hypothesis of BPD postulates impaired alveolarisation as a result of disrupted pulmonary vascular development and states in consequence angiogenesis as the main driver of alveolarisation.^14^ This strongly suggests that the pulmonary development might be highly dependent on the development of the vasculature. Thus, methods are needed to study developmental mechanisms in health and disease by quantitatively investigating the different parts of the pulmonary vasculature. Several studies have analysed the pulmonary vessel tree,^15^, ^16^, ^17^ as well as the ACN,^18^, ^19^, ^20^, ^21^, ^22^, ^23^, ^24^ addressing the identification, quantification or visualisation of morphological alterations caused by BPD. For the ACN, stereological evaluation methods were applied and digital 3D visualisation approaches based on segmentation data were developed in recent years.

Classical stereological parameters characterising the ACN are volume and surface area as those are measures for the gas exchange area and blood volume.^7^ Those are influenced by sample preparation parameters like the perfusion pressure, therefore, the stereological determination of the Euler number χ was established for the ACN by Willführ et al.^18^ This parameter enables comparisons between samples prepared in different experimental settings. The Euler number χ is a measure of connectivity and can be used to estimate the number of septal capillary loops (SCL) in the ACN, which is defined as the shortest cycle of capillary segments around a tissue pillar.^18^, ^22^ It is thus capable of identifying restructuring processes and can be manually obtained by a stereological disector analysis or automatically acquired based on digital ACN segmentations. However, a digital approach was not able to replace the stereological method yet, due to prohibitively time‐consuming segmentation procedures. It was complicated by erythrocytes located within the pulmonary vasculature, which were not included in automatically generated segmentations. Nevertheless, it is crucial to include those blood cells in the segmentation data as erythrocytes that fully block capillaries lead to missing connections within the ACN and an underestimation of the number of SCL. In contrast, erythrocytes that do not fully block the ACN can result in false loops and thus an overestimation of the number of SCL.^18^ Therefore, in the past, the generation of suitable segmentation data even of small parts of the ACN often required months of (manual) work. Thus, only the completely manual stereological determination of the Euler number χ was applied for quantitative ACN studies regarding normal and aberrant lung development.^18^, ^22^, ^23^, ^24^ Grothausmann et al.^19^ established a semiautomated digital watershed filter‐based segmentation method (WSM) to generate 3D reconstructions of lung samples, including the ACN, from light microscopic data. This method is able to offer context for quantitative stereological data with 3D ACN reconstructions.^21^ Nevertheless, previous problems regarding the generation of segmentation data could not be overcome, which in some cases resulted in completely manual segmentation workflows in subsequent studies.^23^ Despite this limitation, the WSM proved to be applicable on serial block‐face scanning electron microscopy (SBF‐SEM) data,^21^ as this imaging technique was evaluated to be suitable to investigate the ACN with an enhanced resolution.^20^

Overall, in recent years, analysis methods for the ACN were firstly enhanced by the application of parameters like the Euler number χ, which provided more detailed quantitative information about the ACN architecture, and can be estimated by classical manual stereological methods.^18^, ^22^ Stereological studies were then supplemented by 3D visualisation approaches, adding qualitative analyses and contextual information based on light or electron microscopic data.^19^, ^20^, ^21^ Regarding the automation of the ACN segmentation process, erythrocytes located within the pulmonary vasculature are still a major challenge. Although tools for automated quantitative analyses of segmentation data were available, they could not be applied on larger parts of the ACN due to the limited segmentation quality.^18^, ^19^

Summarising, the efficiency of digital ACN segmentation approaches is currently low, preventing automated quantitative analyses based on segmentation data with representative sample sizes. Stereological methods, such as the determination of the Euler number χ, allow an efficient quantitative investigation of the ACN. It offers more detailed information than classical stereological parameters alone; however, the information about the ACN lacks context and the data has to be acquired fully manually. An enhanced analysis approach would ideally automatically segment complete lung samples in 3D, which would build the basis for visualisation and further quantitative data acquisition, enabling representative group sizes for future studies. As the central problem of previous studies was the efficient inclusion of erythrocytes into the ACN segmentation, this is a major point that needs to be addressed.

Therefore, in this study, a new workflow approach to more efficiently digitally segment the ACN on SBF‐SEM data was tested, to use the segmentations for 3D visualisation of the ACN, as well as for quantitative automated analyses. The task to be performed is a semantic segmentation^25^, ^26^ that attributes each pixel of the image to a defined category. Here, the relevant category was the lumen of the pulmonary vasculature, which is referred to as the ACN segmentation/label in the following. This category needs to separated from remaining image regions, collectively referred to as the background label. Semantic segmentation is a proven application of machine learning techniques,^25^, ^27^, ^28^ for example, in the form of deep learning (DL).^29^ Thus, this study tested the suitability of a DL‐based segmentation approach for processing SBF‐SEM data to generate ACN segmentations and to perform quantitative automated analyses based on them.

This project was embedded in a larger BPD study. Therefore, already existing SBF‐SEM data of pulmonary blood vessels of an established rabbit BPD model consisting of three animal groups^22^, ^30^, ^31^, ^32^, ^33^, ^34^ was used. In total, three 3D SBF‐SEM datasets were available, which is not a sufficient amount of data to implement a 3D fully generalisable DL‐based segmentation method. Thus, the 2D convolutional artificial neural network U‐Net^35^ was tested as a first step, by interpreting every slice of the 3D datasets as an individual 2D image. The segmentation results were qualitatively and quantitatively evaluated and compared to segmentation results generated by the WSM and to ground truth (GT) segmentation data. Moreover, an automated quantitative ACN analysis determining, for example, the Euler number χ based on segmentations was performed. The results were compared to a manual stereological estimation to test the segmentation quality and reliability of the acquired quantitative data. With this procedure, on the one hand further prospects for the implementation of a DL‐based segmentation method were shown. On the other hand, it was examined if the quantitative analysis approach provided reliable data about the ACN architecture.

## 2 MATERIALS AND METHODS

### 2.1 SBF‐SEM data generation and preprocessing

SBF‐SEM datasets of the pulmonary vasculature were taken from a rabbit BPD model (New Zealand White and Flemish Giant cross‐breed, provided by the Biomedical Sciences animal facility of KU Leuven). The Ethics Committee for Animal Experimentation based at the KU Leuven (Belgium) approved all processes regarding animals of the study (no. 744/2017 and P165/2021). It complied with the European regulations on animal research and the ‘Animal Research: Reporting of In Vivo Experiments’ (ARRIVE) guidelines. The model consisted of a premature hyperoxic (HYX, ≥ 95% atmospheric oxygen concentration), premature normoxic (NOX, 21% atmospheric oxygen concentration) and a term‐born normoxic control group (CO). The CO group was used to distinguish between alterations of the ACN associated with prematurity and hyperoxia (HYX) or prematurity alone (NOX).^22^, ^36^

In the NOX and HYX groups, the rabbit pups were delivered prematurely at 28 of 31 days of gestation by cesarean section. Thereafter, the animals were moved to an incubator with 32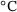 temperature, 50% humidity, and atmospheric oxygen concentrations depending on the assigned animal group. For the CO group, the rabbit pups were term‐born after 31 days, placed in an incubator and exposed to normoxic conditions. After 7 days for the preterm‐born pups and after 4 days for the term‐born rabbits, ensuring identical gestational age, the animals were deeply anaesthetised (35 mg/kg ketamine and 6 mg/kg xylazin) and euthanised. The lungs were perfusion‐fixated with an aldehyde fixative. An airway pressure of 5 cm $H_{2}O$ was maintained, the lung vasculature was perfused with the fixative at a pressure of 25 cm $H_{2}O$ and afterwards the lungs were removed from the body and kept in fixative until further processing.

For the current study, one tissue sample from one animal per treatment group was used. A stereological study of 25 animals from these three treatment groups has been published by Rößler et al.^22^ The preparation of small blocks of the lungs for SBF‐SEM imaging was conducted identically for all samples.^20^, ^37^, ^38^ As part of this procedure, the fixated tissue blocks were stained using a reduced osmium tetroxide, thiocarbohydrazide, osmium tetroxide (rOTO) protocol with uranyl acetate and lead aspartate for contrast and conductivity enhancement. After dehydration, the samples were embedded (Durcupan^TM^ ACM resin, Sigma‐Aldrich, St. Louis, USA), placed using conductive epoxy glue (Chemtronics, CircuitWorks, Kennesaw, USA) on a specimen pin (Gatan, Pleasanton, CA, USA) and sputter coated with a gold layer of 20 nm thickness. For the imaging process a Zeiss Merlin VP Compact scanning electron microscope (SEM, Carl Zeiss Microscopy GmbH, Jena, Germany) with a Gatan 3View2XP system (Gatan Inc., Pleasanton, CA, USA) was used. Imaging was performed with 3 kV acceleration voltage and a dwell time of 0.7 µs in the variable pressure mode at 30 Pa. For each animal group, a single 3D SBF‐SEM dataset was generated. Thus, in total three SBF‐SEM datasets were available. The associated dimensions and resolutions are listed in Table 1.

**TABLE 1:** Dataset overview for the NOX, HYX and CO samples of the rabbit BPD model. dim.: dimension (voxels in the dataset), res.: resolution (physical space captured in one voxel), WSM: watershed filter‐based segmentation method, MAN: manual segmentation, DL: deep learning‐based segmentation approach.

	**NOX**	**HYX**	**CO**
**Raw data dim**. [vx]	10,000 × 10,000 × 7500	10,000 × 10,000 × 7500	10,000 × 10,000 × 7500
**Raw data res**. [nm]	40 × 40 × 80	40 × 40 × 80	40 × 40 × 80
**Preprocessed data dim**. [vx]	1866 × 1866 × 3998	1866 × 1866 × 3999	2466 × 2466 × 3997
**Preprocessed data res**. [nm]	150 × 150 × 150	150 × 150 × 150	150 × 150 × 150
**Segmented data dim**. [vx]	1866 × 1866 × 3800	1866 × 1866 × 3999	2466 × 2466 × 3000
**Segmented data res**. [nm]	150 × 150 × 150	150 × 150 × 150	150 × 150 × 150
**Segm. methods: WSM, MAN** [vx]	1866 × 1866 × 2800	1866 × 1866 × 3999	−
**Segm. methods: DL, WSM, MAN** [vx]	1866 × 1866 × 1000	−	2466 × 2466 × 3000
**Number of images for training data**	1800	3399	−
**Segm. methods**	WSM, MAN	WSM, MAN	−
**Number of images for test data**	−	−	2000
**Analysed data dim**. [vx]	1000 × 1000 × 3000	1000 × 1000 × 3000	1000 × 1000 × 3000
**Analysed data res**. [nm]	150 × 150 × 150	150 × 150 × 150	150 × 150 × 150

The SBF‐SEM data preprocessing procedure was identical for all three datasets and involved resampling to an isotropic voxel size of 150 nm, which is sufficient to resolve the microvasculature of the lung^20^ and reduced the dataset size and thus memory requirements. This was done using the Insight Toolkits (ITK)^39^ ‘LinearInterpolateImageFunction’. Contrast adjustment was performed slice‐wise to compensate for variations that emerged over the acquisition time. Due to shifts in *x*‐ and *y*‐direction caused by the acquisition process, the datasets had to be aligned after stacking the individual image slices along the *z*‐axis, using the ImageJ plugin ‘Linear Stack Alignment with SIFT’.^40^ Resulting edges were cropped afterwards. This led to different dataset dimensions for the NOX/HYX and CO samples due to differences in the occurring shifts. Single slices showing strong artifacts, were rejected. The final dimensions of the preprocessed datasets can be found in Table 1.

### 2.2 Segmentation of NOX and HYX SBF‐SEM datasets

**FIGURE 1:**
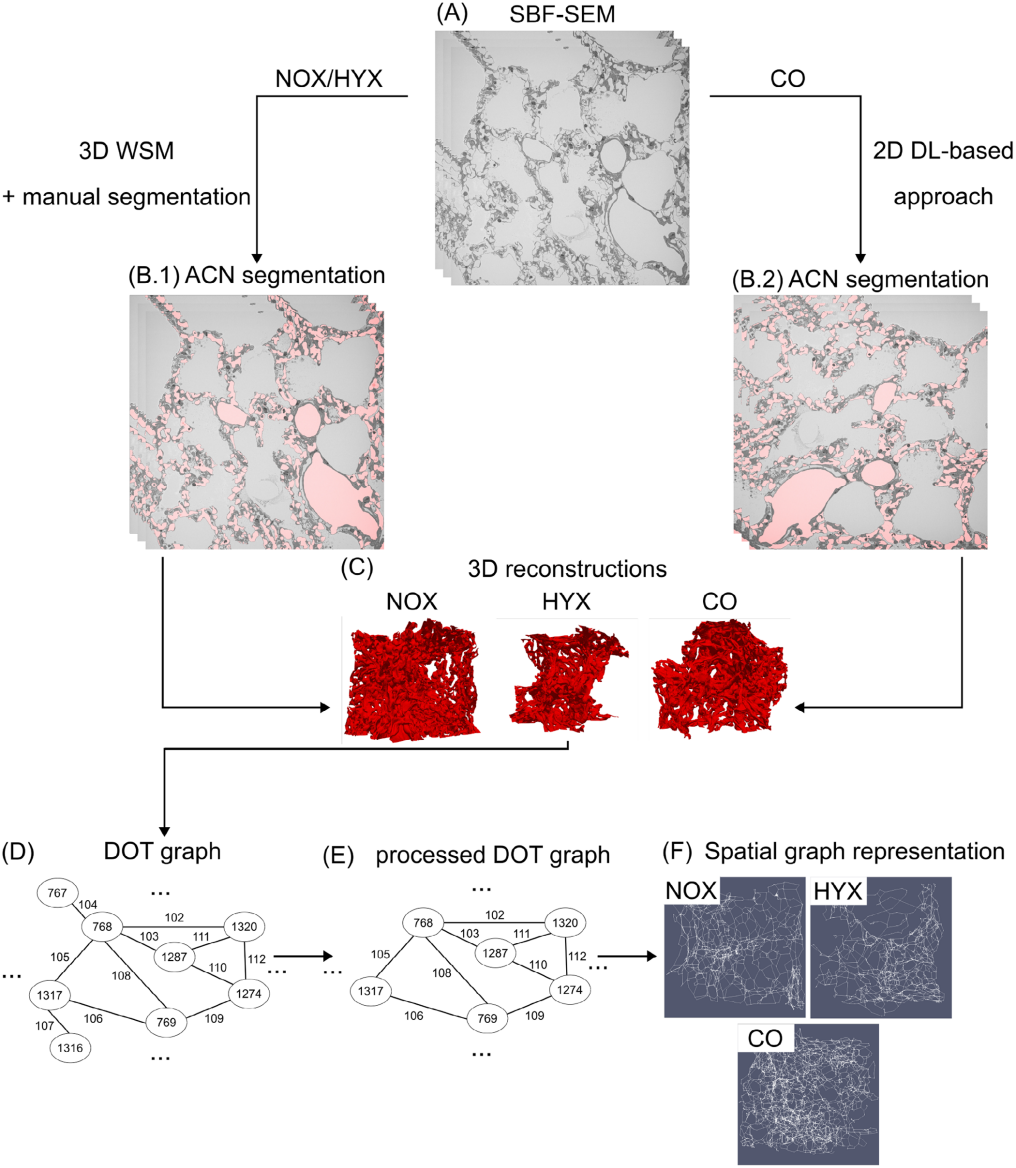
Workflow overview of the digital ACN segmentation based on SBF‐SEM data, 3D reconstruction and analysis. (A) For each animal group (NOX, HYX, CO) one 3D SBF‐SEM dataset was available. (B.1) The NOX and HYX datasets were segmented using the established WSM, combined with manual segmentation. (B.2) The CO dataset was processed testing a DL‐based approach. (C) Based on the segmentations, 3D models of the ACN surface were reconstructed for all available datasets. (D) Processing of the 3D models to analysable DOT graphs consisting of nodes and edges. (E) Postprocessing of the DOT graphs by deleting nodes with only one neighbour. (F) The DOT graphs were translated into a spatial graph representation of the ACN.

**FIGURE 2:**
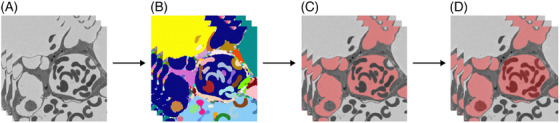
Workflow overview of the 3D WSM to generate ACN segmentations based on SBF‐SEM data (A) for the NOX and HYX samples. (B) A 3D watershed filter was used to generate a presegmentation containing all individual labels (shown in different colours) for identified connected objects. (C) Based on the presegmentation, an initial segmentation was generated containing the largest continuous ACN label (transparently overlaid in red on the SBF‐SEM data). (D) Afterwards, manual segmentation work was conducted to complete the ACN segmentation, that is, the red ACN label now also covers cells and and other spaces previously not included in this segmentation label. Different backgrounds lead to different intensities of the transparent red label. This does not represent additional class labels.

**FIGURE 3:**
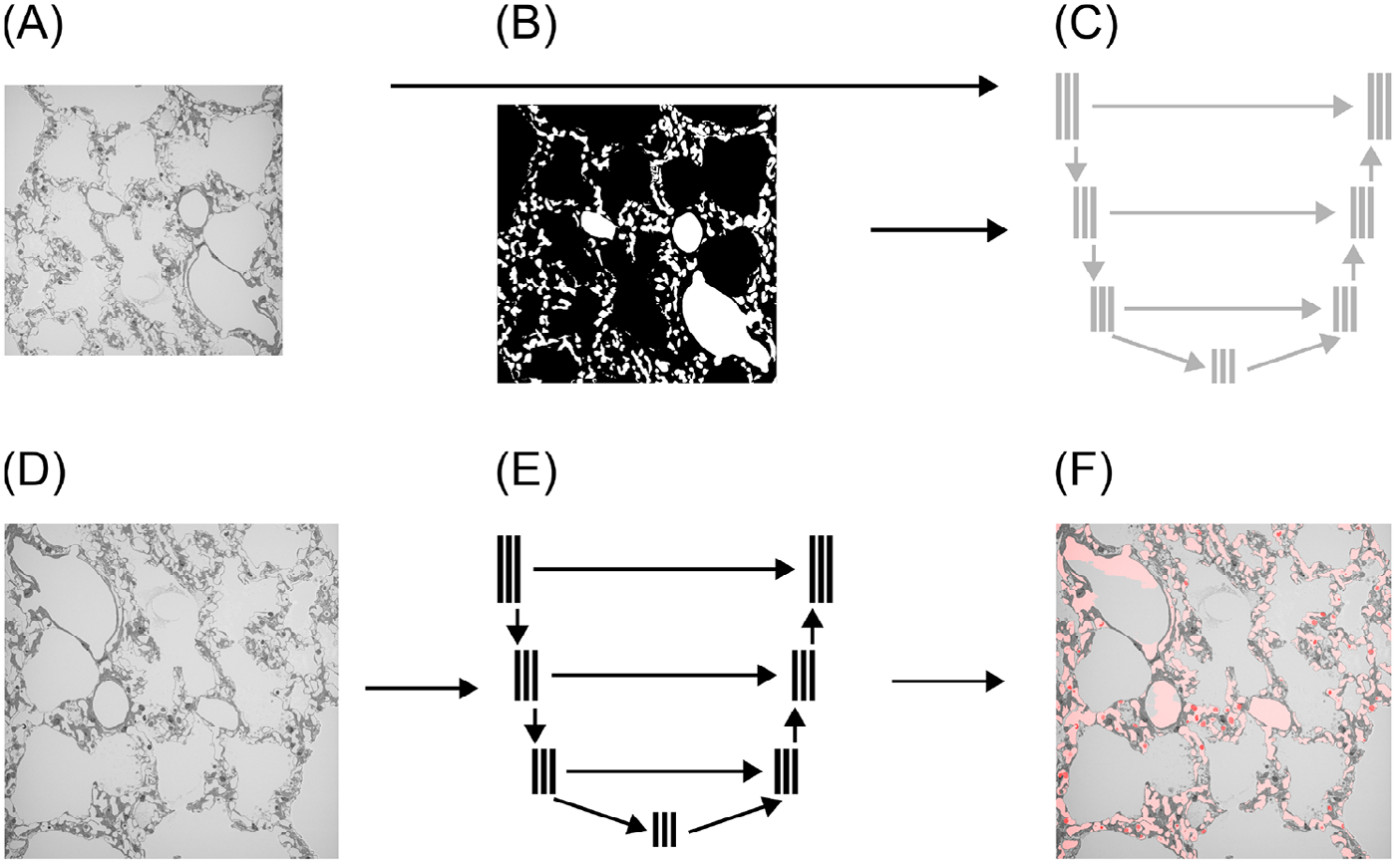
Workflow overview of the test of a DL‐based segmentation approach. (A) Existing SBF‐SEM datasets with corresponding ACN segmentations (B) of the NOX and HYX samples were used as training data by interpreting slices of the 3D datasets as individual 2D images. (C) With them, the 2D artificial neural network was trained. The trained artificial neural network (E) was applied on extracted 2D SBF‐SEM images of the CO sample (D), which was used as test data to generate ACN segmentations (F) for the individual 2D slices.

A general workflow overview is given in Figure 1. The SBF‐SEM datasets of the NOX and HYX samples were segmented using the established WSM (Figure 1B.1) described by Grothausmann et al.^19^ A detailed workflow overview of the WSM can be found in Figure 2. A 3D watershed filter^41^, ^42^, ^43^ was applied to the preprocessed SBF‐SEM datasets (Figure 2A). The watershed filter categorised image areas according to their grey value and a global manually set threshold, resulting in the generation of different labels for separated image areas in 3D. This process generated a presegmentation (Figure 2B) that contained all generated labels. A low threshold resulted in so‐called oversegmentation, where connected structures in the SBF‐SEM data were fragmented into multiple labels in the presegmentation. A higher threshold reduced the number of labels in the presegmentation. However, if the threshold was chosen too high, undersegmentation occurred. In that case, borders of structures in the SBF‐SEM data that needed to be preserved got covered by the generated labels. This would connect the ACN label with the adjacent extensive label of the air‐space, which needs to be prevented to ensure that the ACN segmentation represents the biological structures. In this study, the watershed filter threshold was chosen by iterative adjustment, until the maximum was reached, before undersegmentation occurred. Local oversegmentation could not be avoided in that case across the dataset, as can also be seen in the example area in Figure 2B. Subsequently, an initial segmentation was generated by assigning the largest continuous label located within the vasculature contained in the presegmentation, manually to the ACN label (Figure 2C). Next, small holes in the initial segmentation that were fully enclosed by the ACN label were filled using the ITK ‘BinaryFillHoleImageFilter’ (BFH‐filter)^44^, ^45^, ^46^ as part of a postprocessing procedure. Additionally, manual segmentation work was performed by digitally adding parts of the vasculature contained in the presegmentation to the ACN label, using a special version of ITK‐SNAP (https://github.com/pyushkevich/itksnap/pull/1). Here, especially the manual segmentation of blood cells like erythrocytes located in the ACN was a highly time‐consuming factor, as described in previous studies.^18^, ^19^, ^20^, ^23^ Necessary further manual corrections of the segmentation were executed using the ITK‐SNAP paintbrush tool to generate a continuous as possible and reliable ACN segmentation (Figure 2D). As a final step, the ITK BFH‐filter was applied again. In this way, 2800 out of 3998 2D images of the 3D NOX SBF‐SEM dataset and all slices of the HYX dataset were segmented (see Table 1). The entire NOX dataset could not be segmented with sufficient quality due to factors like blood cells located within the pulmonary vasculature. Parts of the segmentations with low quality were manually identified and excluded. The generated segmentations were on one hand used to test an automated quantitative ACN analysis approach (Figure 1C–F) and on the other hand, in combination with the SBF‐SEM data, for the training procedure of a DL‐based segmentation approach (Figure 3A).

### 2.3 Test of a DL‐based approach for segmenting the ACN on SBF‐SEM data

For this study, the amount of available data for testing a DL‐based segmentation approach was limited. Therefore, a 2D approach using the artificial neural network U‐Net,^35^ implemented in an established project (https://github.com/milesial/Pytorch‐UNet) was tested to segment the ACN on SBF‐SEM data (Figure 1B.2). A training procedure was conducted using segmented SBF‐SEM data of the NOX and HYX samples, as described in Section 2.2. Therefore, from the 3D SBF‐SEM datasets, 2D images were extracted and used in combination with the corresponding ACN segmentations of the individual slices for the training procedure (Figure 3A). 1800 2D images from the NOX sample and 3399 2D images from the HYX sample (see Table 1) were included in the training procedure (Figure 3B). No additional data augmentation was conducted. Training was performed on a CPU (Intel Xeon E5‐2667 v4 @ 3.20 GHz processor), with 10% validation data, a downscaling factor of 0.75 (bicubic resampling for images and nearest neighbour for masks), a learning rate of $e^{-}5$, and completed after 10 epochs. After every epoch, the trained network was saved. A trained network for the following tests was chosen based on the training and validation loss which were monitored during the training procedure. At epoch 4, both training and validation loss were low, before an increase was identified, starting at epoch 6. Therefore, the trained network at epoch 4 was used for further tests in this study, to prevent overfitting as good as possible.

The trained network (Figure 3C) was applied to CO SBF‐SEM test data to segment the ACN. Here, again 2D images of the 3D CO SBF‐SEM dataset were extracted and individually processed with the trained artificial neural network (Figure 3D and E). Following, the ITK BFH‐filter was applied. In this way 2000 2D SBF‐SEM slices extracted from the 3D CO dataset were segmented as test data (see Table 1).

To assess the suitability of the DL‐based approach to segment the ACN, qualitative and quantitative evaluations were carried out. Therefore, the DL‐based results for the test data were compared to segmentation results generated by the WSM for identical SBF‐SEM data. Thus, for the WSM the status of the initial segmentation (Figure 2C) was used for comparison, which was generated with minimal manual intervention. The segmentation results of the DL‐based approach and WSM were therefore only postprocessed using the ITK BFH‐filter. No manual segmentation or correction work was performed to ensure comparability of the approaches. The ACN segmentation results were qualitatively compared regarding three evaluation criteria:

1. Segmentation of blood cells like erythrocytes located within the pulmonary vasculature,
2. Segmentation of pre‐ or postcapillary blood vessels,
3. Excessive segmentation of the ACN label (false‐positive segmentation, undersegmentation).

For the quantitative evaluation of the initial segmentation results, the segmentations generated by the DL‐based approach and the WSM were compared to GT segmentation data using established metrics. The GT data aimed to represent a segmentation status that was as complete as possible, which was manually checked. For the test data (CO sample), GT segmentations were generated by firstly applying the trained neural network to the 2D slices extracted from the 3D SBF‐SEM dataset. This segmentation status was used as an initial segmentation and was manually extended. Finally, the BFH‐filter was applied. Quantitative performance evaluation of the segmentation approaches was carried out using the metrics of sensitivity (SEN), specificity (SPE), positive predictive value (PPV), negative predictive value (NPV) and Dice similarity coefficient (DSC). The generated ACN segmentations by the DL‐based approach and the WSM were compared pixel‐wise for each slice to the GT data, classifying each pixel of the segmentation image as true positive (TP), true negative (TN), false positive (FP) or false negative (FN) as defined in Table 2.^47^, ^48^ In this case, ‘positive’ (pixel value of 1 in Table 2) referred to a pixel categorised to the ACN label. Thus, a TP pixel would be a pixel located within the pulmonary vasculature on the SBF‐SEM data that was correctly categorised to the ACN label on the segmentation data. A FP pixel would be a pixel located outside the pulmonary vasculature that was incorrectly categorised to the ACN label. ‘Negative’ (pixel value of 0 in Table 2) referred to a pixel categorised to the background label. A TN pixel would be a pixel located outside the pulmonary vasculature that was correctly categorised to the background label and a FN pixel would be located inside the pulmonary vasculature, incorrectly categorised to the background label. Based on the number of pixels (n) categorised as TP, TN, FP and FN for each analysed slice, the evaluation metrics were calculated as follows:

(1) $$\begin{array}{c} SEN=\frac{nTP}{nTP+nFN}, \end{array}$$

(2) $$\begin{array}{c} SPE=\frac{nTN}{nTN+nFP}, \end{array}$$

(3) $$\begin{array}{c} PPV=\frac{nTP}{nTP+nFP}, \end{array}$$

(4) $$\begin{array}{c} NPV=\frac{nTN}{nTN+nFN}, \end{array}$$

(5) $$\begin{array}{c} DSC=\frac{2\cdot nTP}{2\cdot nTP+nFP+nFN}. \end{array}$$

**TABLE 2:** Definition of true positive (TP), true negative (TN), false positive (FP), false negative (FN) for binary image data. ‘Positive’ (1) refers here to a pixel categorised as the ACN label. ‘Negative’ (0) refers to a pixel categorised as the background label. GT: ground truth, SEG: segmentation.

	***TP***	***TN***	***FP***	***FN***
**GT**	1	0	0	1
**SEG**	1	0	1	0

The parameters were averaged over all slices in the end, resulting in one value per parameter for both the WSM and DL‐based segmentation approach. The parameter SEN was in this case a measure for the completeness of the ACN label, whereas the parameter SPE was a measure for the completeness of the background label. The PPV was the fraction of TP pixels in the segmentation data out of all pixels categorised as the ACN label. The NPV was defined as the fraction of TN pixels in the segmentation data out of all pixels categorised as the background label. The DSC functions as a measure of the overlap of two images. It reaches its maximum at 1, indicating identical images.^25^, ^47^, ^48^

### 2.4 Automated quantitative ACN analysis

Out of each segmented dataset (NOX, HYX, CO), three subregions with a volume of 1000 × 1000 × 1000 vx (150 × 150 × 150 $\mu m^{3}$) each were extracted for the automated quantitative ACN analysis (see Table 1). This step was necessary, as the segmentations were checked manually regarding the completeness of the ACN label before performing the analysis to exclude regions with low segmentation quality that could potentially lead to unreliable quantitative results about the ACN architecture. For the NOX sample 2000 slices of the ACN segmentation data generated by the application of the WSM in combination with manual segmentation work, as described in Subsection 2.2, were used (see Table 1). For dataset regions that could not be segmented in this way with a sufficient quality, the DL‐based approach was applied to slices that were excluded from the training procedure of the DL artificial neural network. Afterwards, manual segmentation work was performed based on a presegmentation generated by the WSM and the utilisation of the ITK‐SNAP paintbrush tool. 1000 slices of the dataset were segmented using this procedure (see Table 1). For the HYX sample, the segmentations of the generated training data were used to conduct the automated ACN analysis (3000 slices). For the CO sample, the GT data (2000 slices) was used for the analysis. Using the same segmentation procedure, ACN labels for 1000 additional slices were generated (see Table 1). The segmentations for all subregions were postprocessed by removing unconnected parts of the ACN label in 3D, resulting in one connected ACN segmentation per subregion.

For each of the nine subregions, a 3D model of the ACN was reconstructed by stacking the segmentation data along the *z*‐axis. It was ensured, that all 1000 slices per volume were consecutive, with no gaps in between. Consequently, three ACN reconstructions per dataset were generated from one available sample per animal group. To visualise the results, the surface mesh of the ACN was exported using ITK‐SNAP^44^, ^45^ and displayed using ParaView^49^ (Figure 1C). In the next step, the 3D ACN segmentations were processed to DOT graphs using the ‘Spatial Graph Extractor’ (SGEXT) tool (https://github.com/phcerdan/SGEXT) (Figure 1D), which are analysable using automated software tools.^16^ The generated graphs consisted of nodes connected by edges,^50^ both assigned individual IDs. Nodes represented branching points of the ACN, edges represented capillary segments. The graphs were postprocessed by deleting nodes with only one neighbour and adjacent branching edges (Figure 1E), since these nodes represented regions that are not biologically meaningful.^18^ These dead‐ends were possibly present due to artefacts caused by the shrinking process of the segmentation data or sample boundaries. Finally, the graphs were translated into 3D space, visualising the skeleton and the network structure of the ACN using ParaView (Figure 1F).^16^, ^17^

The DOT graphs were automatically analysed using a Python script (https://github.com/schmidt‐julia/acn_analysis.git), employing the Python NetworkX library.^51^ The first analysis step was to obtain the number of nodes $N_{\text{nodes}}$ and edges $N_{\text{edges}}$ for all subregions using integrated NetworkX functions. With the number of nodes and edges, the Euler number $\chi_{\text{graph}}$ of the ACN subregions was determined using Equation (6) ^18^:

(6) $$\begin{array}{c} \chi_{\text{graph}}=N_{\text{nodes}}-N_{\text{edges}}. \end{array}$$

With $\chi_{\text{graph}}$ the number of SCL $N_{\text{SCL, graph}}$ in the ACN subregions could be determined using Equation (7):

(7) $$\begin{array}{c} N_{\text{SCL, graph}}=|\chi_{\text{graph}}|+1. \end{array}$$

As a SCL is defined as the shortest cycle of capillary segments around a tissue pillar in the ACN,^18^, ^22^ it was expected to be equivalent to the minimal number of polygons $N_{\text{SCL, polygon}}$ (i.e., the cycle basis^50^) required to form the network $N_{\text{SCL, graph}}$. Thus, the cycle basis of each subregion was identified using an integrated NetworkX function.^52^ The number of SCL $N_{\text{SCL}}$ was obtained by these two methods and the results were compared to verify that they are indeed identical.

To generate quantities with similar units for comparison with results acquired by a manual stereological estimation, the numerical density of SCL $N_{\text{V, polygon}}$ was determined by dividing the number of polygons $N_{\text{SCL, polygon}}$ by the image volume V (Equation 8). In this case the volume V of each analysed ACN subregion was equal to 150 µm^3^.

(8) $$\begin{array}{c} N_{\text{V, polygon}}=\frac{N_{\text{SCL, polygon}}}{V}. \end{array}$$

For each dataset, all three subregions were analysed. The obtained results were averaged per dataset and compared between the CO and NOX sample and the NOX and HYX sample by calculating the percentage deviation, as this would be the preferred proceeding to reveal ACN changes due to prematurity and hyperoxia or prematurity alone if a larger sample size was available, like in Rößler et al.^22^ In the present study, the sample size was limited; therefore, it was not expected to generate representative results about BPD‐induced ACN alterations. Additionally, the percentage deviations between the CO and HYX samples were calculated to compare the results.

### 2.5 Quantitative stereological estimation

The automatically obtained quantitative data about the ACN architecture was compared in a two‐step process to results of a stereological estimation. Thus, stereological data of the numerical density of SCL $N_{\text{V, stereol.}}$ was acquired manually for both the preprocessed SBF‐SEM data and the postprocessed ACN segmentations of the same ACN subregions that were automatically analysed.

First, the stereological estimation of the numerical density of SCL obtained from SBF‐SEM data $N_{\text{V, stereol. EM}}$ was compared to stereological data acquired from the segmentations $N_{\text{V, stereol. seg.}}$ to assess the segmentation quality. Second, the reliability of the automatically obtained data was assessed by comparing $N_{\text{V, stereol. EM}}$ to the numerical density of SCL based on the identified number of polygons that the network is composed of $N_{\text{V, polygon}}$. The comparison was conducted between $N_{\text{V, polygon}}$ and $N_{\text{V, stereol. EM}}$ (not $N_{\text{V, stereol. seg}}$), as a stereological method would use SBF‐SEM and not segmentation data for the determination of the numerical density of SCL. Therefore, the automatically determined results should reproduce the data obtained based on SBF‐SEM data to provide a reliable impression of the ACN architecture. $N_{\text{V, stereol. seg.}}$ was obtained in this study only to assess the segmentation quality.

To determine $N_{\text{V, stereol.}}$, a disector analysis was performed. For this task, a disector height $h_{\text{dis}}$ of 1 µm and a counting frame with an area $a_{\text{frame}}$ of 22,500 m^2^ were chosen. First, the Euler number $\chi_{\text{stereol.}}$ was determined using the Euler‐Poincaré characteristic by counting the stereological events called bridges and islands on two consecutive dataset sections. Bridges are defined as new connections between capillaries, islands as emerging new capillary profiles when comparing the disector slices. For each analysed subregion more than 100 counting events were registered and with the number of islands and bridges, the Euler number $\chi_{\text{stereol.}}$ was determined as defined in Equation (9).^18^, ^53^

(9) $$\begin{array}{c} \chi_{\text{stereol.}}=\sum \text{islands}-\sum \text{bridges}. \end{array}$$

The numerical density of SCL $N_{\text{V, stereol.}}$ was determined by relating the Euler number $\chi_{\text{stereol.}}$ to the disector volume using Equation 10 for each subregion. The number of analysed counting frames is described by $\sum n_{\text{par}}$, the area of a counting frame is described by $a_{\text{frame}}$ and the disector height by $h_{\text{dis}}$. The multiplication by two is necessary to compensate for counting events on both images of the disector. In the end, the results for each subregion were averaged per dataset.

(10) $$\begin{array}{c} N_{\text{V, stereol.}}=\frac{-\chi_{\text{stereol.}}}{2\cdot \sum n_{\text{par}}\cdot a_{\text{frame}}\cdot h_{\text{dis}}}. \end{array}$$

## 3 RESULTS

### 3.1 Qualitative ACN segmentation evaluation

**FIGURE 4:**
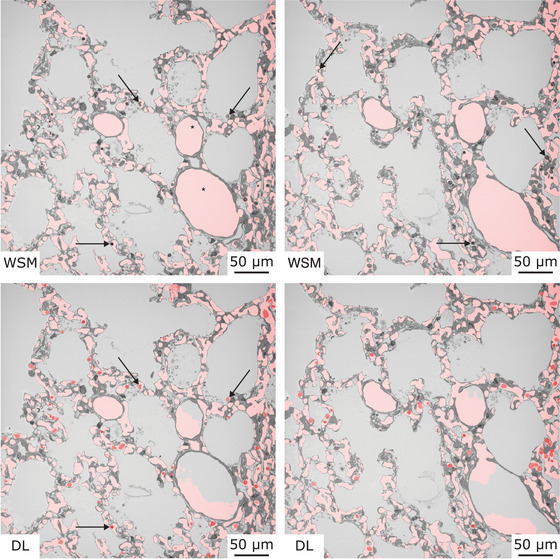
Initial segmentation results of the WSM and the DL‐based segmentation approach. Two individual slices of SBF‐SEM test data transparently overlaid with ACN segmentation results (red) of the WSM (top row) and the DL‐based segmentation approach (bottom row) are shown. Images in the same column show identical SBF‐SEM images. Qualitative differences in the results of the WSM and DL‐based segmentation approach regarding the segmentation of blood cells located within the pulmonary vasculature (exemplified highlighted by arrows) and of pre‐ or postcapillary blood vessels (highlighted by asterisks) are noticeable.

In Figure 4, segmentation results of the WSM (top row) and the DL‐based segmentation approach (bottom row) applied to CO SBF‐SEM test data are exemplified for two individual slices. Here, an overview of the achieved ACN segmentation results, which are transparently overlaid in red, is provided. Images in the same column show identical SBF‐SEM images. In this figure, the qualitative differences in the segmentation of blood cells located within the pulmonary vasculature (exemplified highlighted by arrows) and the segmentation of pre‐ or postcapillary blood vessels (highlighted by asterisks) are particularly striking. Blood cells located within the pulmonary vasculature were not included in the segmentation of the WSM, as expected. In contrast, these image parts were correctly categorised by the DL‐based segmentation approach, despite the use of a 2D approach and the limited amount of training data. Contrary results were obtained for the segmentation of pre‐ or postcapillary blood vessels, as the WSM delivered more complete segmentation results regarding this qualitative evaluation criterion.

**FIGURE 5:**
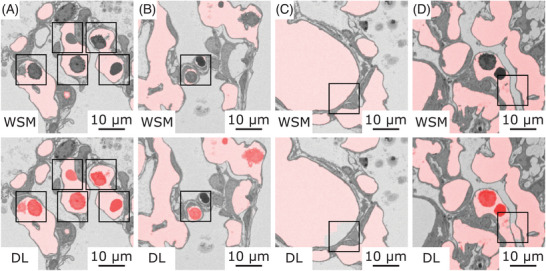
CO SBF‐SEM sections of test data, transparently overlaid with initial ACN segmentation results (red) of the WSM (top row) and the DL‐based segmentation approach (bottom row). Areas regarding the qualitative evaluation criteria are highlighted by black boxes. Images in the same column show identical SBF‐SEM image sections. (A) The segmentation of blood cells like erythrocytes located within the pulmonary vasculature. (B) The segmentation of blood cells inside capillaries. (C) Segmentation results of pre‐ or postcapillary blood vessels. (D) Excessive segmentation of the ACN label (false‐positive segmentation).

All qualitative evaluation criteria were examined in more detail in Figure 5. In this figure, WSM segmentation results are shown for four image subsections (Figure 5A–D) in the top row, and the DL‐based segmentation results for the same image subsections in the bottom row. Figure 5A shows the segmentation of blood cells located within the pulmonary vasculature. Parts highlighted by black boxes in the top row were incorrectly assigned to the background label in the segmentation results of the WSM. The missing labels would have to be manually added in the next step to the ACN label to generate a complete segmentation. In comparison, the DL‐based segmentation approach generated more complete segmentation results regarding this qualitative evaluation criterion (Figure 5A, bottom row). In Figure 5B, examples for the segmentation of blood cells located within capillaries are shown. Image parts highlighted by black boxes show capillaries where nearly the whole lumen is filled by blood cells. With the WSM, the blood cells were not included in the ACN segmentation result, as expected. The segmentation results of the DL‐based segmentation approach were more complete, but limitations of the 2D segmentation approach became visible as incorrect segmented parts appeared when the lumen area around the blood cell decreased, as can be seen in Figure 5B in the bottom row. Figure 5C shows segmentation results of pre‐ or postcapillary blood vessels, which should be continuously assigned to the ACN label. The WSM delivered complete segmentation results for this scenario, as can be seen in the top row. The DL‐based segmentation approach did not deliver continuous segmentation results regarding this criterion at this point, as can be seen in Figure 5C in the bottom row. The last qualitative evaluation criterion, was the excessive segmentation of the ACN label (false‐positive segmentation). For the applied WSM, the case of undersegmentation only occurred when the global watershed filter threshold was chosen too high. In the top row of Figure 5D, it can be seen that false‐positive segmentation was prevented in the ACN segmentation results for the example image section. For the DL‐based segmentation approach, false‐positive segmentation was found in the ACN segmentation results, highlighted by the black box in Figure 5D in the bottom row.

As a first result it was qualitatively shown that the two segmentation approaches delivered diverging ACN segmentation results. The DL‐based segmentation approach seemed to be especially advantageous regarding the segmentation of blood cells located within the pulmonary vasculature. For this situation, the WSM has reached its limits due to the application of a global threshold. The WSM delivered better segmentation results regarding continuously enclosed objects, like pre‐ or postcapillary blood vessels, with less false‐positive segmentation when the watershed filter threshold was appropriately chosen. At this point, both segmentation approaches need manual segmentation and correction work to generate a complete ACN segmentation.

### 3.2 Quantitative ACN segmentation evaluation

For a quantitative assessment of the segmentation performance of both approaches, the evaluation parameters described in Subsection 2.3 were calculated (Equations (1), (2), (3), (4), (5)), averaged and the results were listed in Table 3 with associated standard deviations (SD).

**TABLE 3:** Quantitative evaluation of the performance of both segmentation approaches. The initial segmentation results of the WSM/DL‐based segmentation approach and GT segmentation data were compared for 2000 extracted 2D slices of the CO test dataset. Averaged evaluation metrics of SEN, SPE, PPV, NPV and DSC (Equations (1), (2), (3), (4), (5)) with associated standard deviations (SD) were calculated.

	***SEN*** **(SD)**	***SPE*** **(SD)**	***PPV*** **(SD)**	***NPV*** **(SD)**	***DSC*** **(SD)**
**WSM**	0.8895(0.0659)	1.0000(0.0001)	0.9998(0.0008)	0.9829(0.0064)	0.9401(0.0382)
**DL**	0.9316(0.0602)	0.9958(0.0031)	0.9770(0.0145)	0.9856(0.0177)	0.9527(0.0340)

Overall, the results of SEN, NPV and DSC for the DL‐based segmentation approach outperformed the results of the WSM. For the DL‐based segmentation approach, the parameter SEN indicated a more complete ACN segmentation than using the WSM. The WSM generated a more complete background label, as indicated by the parameter SPE. The differences in the completeness of the labels were larger for the ACN label. The parameter SEN confirmed the qualitative results for the WSM of excluding blood cells located within the pulmonary vasculature from the ACN label. In addition, the qualitative results for the incomplete segmentation of pre‐ or postcapillary blood vessels for the DL‐based segmentation approach were confirmed, as the parameter SEN was below 1. In general, the parameters of SEN and SPE showed for both segmentation methods, that at this point manual segmentation and correction work is necessary and that the DL‐based segmentation approach delivered quantitatively more complete ACN segmentation results.

The parameter PPV, which was higher for the WSM, confirmed the results of the parameter SPE. More than 99% of pixels categorised to the ACN label were correctly assigned by the WSM. In reverse, less than 1% of the pixels assigned to the ACN label were categorised to the false category, reflecting the correct choice and limited possibilities of adjustment for the global watershed filter threshold. The PPV for the DL‐based segmentation approach showed the appearance of more excessive segmentation of the ACN label, compared to the WSM, confirming the qualitative results.

The parameter NPV was higher for the DL‐based segmentation approach, emphasising the results for the parameter SEN. For the DL‐based segmentation approach, more than 98% of the pixels categorised to the background label were correctly assigned, reflecting the qualitative results of the segmentation of pre‐ or postcapillary blood vessels. The lower values for the WSM once again confirmed the qualitative segmentation results and the missing segmentation of blood cells located within the pulmonary vasculature.

Overall, the qualitative results were confirmed by the quantitative evaluation. The size of the impact of the qualitative evaluation criteria on the overall segmentation performance could be estimated. The lower parameter SEN and NPV for the WSM reflected the missing segmentation of blood cells located within the pulmonary vasculature. For the DL‐based segmentation approach, the higher incidence of false‐positive segmentation was confirmed by lower parameters of SPE and PPV. The incomplete segmentation of pre‐ or postcapillary blood vessels and false‐positive segmentation by the DL‐based segmentation approach seemed to have a lower impact on the overall segmentation results than the missing segmentation of blood cells located within the pulmonary vasculature by the WSM, as the parameters of SEN and NPV were higher for the DL‐based segmentation approach. Also, the DSC was overall higher for the DL‐based segmentation approach, verifying this impression. The standard deviation was for most of the parameters lower for the WSM, indicating a higher heterogeneity of the DL‐based segmentation results across the test dataset.

### 3.3 Qualitative and quantitative ACN morphology analysis

**FIGURE 6:**
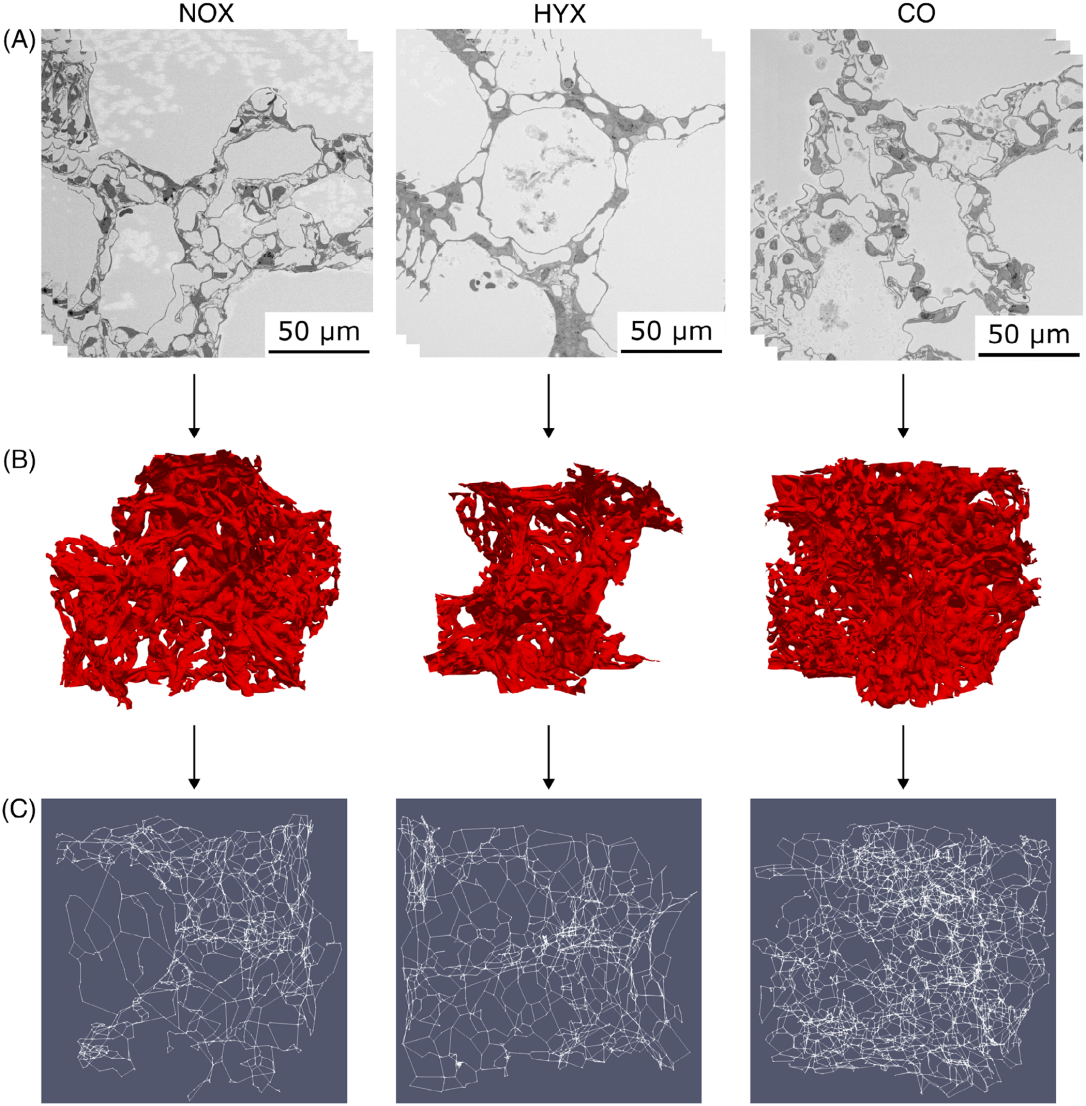
Exemplary visualisation of subregions of the ACN for the NOX, HYX and CO datasets. (A) Exemplary SBF‐SEM slices. (B) 3D reconstruction of the ACN surface based on ACN segmentation data. (C) Spatial graph representations of the ACN.

In Figure 6A–C exemplary SBF‐SEM data, reconstructed 3D models of the ACN surface of one subregion per dataset, and corresponding graphs, translated into 3D space are shown. A qualitative analysis of the SBF‐SEM data revealed remarkable differences between the CO/NOX and HYX samples (Figure 6A). In particular, a reduction of the number of tissue pillars, as well as a reduction in the complexity and density of the microvasculature, were observable. No qualitative differences were observed comparing the CO and NOX samples.

Using the 3D ACN reconstructions and graphs, the most substantial differences were qualitatively observed between the CO and HYX samples (Figure 6B and C). Comparing the NOX and HYX samples, the second biggest differences were noticed. For the HYX sample, a reduction of the density was identifiable. Here, also differences between the CO and NOX datasets were identified that were not observable using only SBF‐SEM data. For the NOX sample, the ACN appeared to be less dense in the 3D reconstruction and graph than the ACN of the CO dataset. This was not qualitatively identifiable based on the SBF‐SEM image stack alone. Quantitative data about the ACN architecture were collected to quantify those differences between the datasets.

Automatically obtained quantitative analysis results based on ACN segmentation data, averaged per dataset, and associated standard deviations (SD) are listed in Table 4 (upper part). In this study, the NOX sample showed a 43.66% decrease of the number of nodes $N_{\text{nodes}}$ compared to the CO sample. Comparing the NOX and HYX samples, a decrease of 16.83% was detectable. The number of edges $N_{\text{edges}}$ was decreased by 43.61% comparing the CO and NOX samples. The HYX sample showed a 13.66% decrease compared to the NOX sample. Consequently, the biggest differences were identified between the CO and HYX samples, regarding the number of nodes $N_{\text{nodes}}$ and edges $N_{\text{edges}}$.

**TABLE 4:** Automated quantitative analysis results of the ACN for the NOX, HYX and CO datasets based on ACN segmentation data (upper part) and stereologically estimated results based on SBF‐SEM (EM) and segmentation data (seg), as well as determined deviations (bottom part). For each dataset three segmented subregions of the ACN were analysed and the results were averaged per dataset. The results were compared between the datasets for parameters of number of nodes $N_{\text{nodes}}$, edges $N_{\text{edges}}$, SCL $N_{\text{SCL, graph}}$ and polygons $N_{\text{SCL, polygon}}$ and the numerical density of SCL $N_{\text{V, polygon}}$. Associated standard deviations (SD) are listed. The results were compared between each other ($N_{\text{V, stereol. EM}}/N_{\text{V, stereol. seg}}$), as well as to the automatically determined numerical densities of SCL based on the number of polygons $N_{\text{V, polygon}}$ ($N_{\text{V, polygon}}/N_{\text{V, stereol. EM}}$) for all datasets and the deviations in percentage were calculated.

	**NOX**	**HYX**	**CO**	**CO/NOX**	**NOX/HYX**	**CO/HYX**
$N_{\text{nodes}}$ **(SD)**	1884(142)	1567(283)	3344(703)	−43.66%	−16.83%	−53.14%
$N_{\text{edges}}$ **(SD)**	2373(174)	2049(391)	4208(895)	−43.61%	−13.66%	−51.31%
$N_{\text{SCL, graph}}$ **(SD)**	490(48)	483(108)	865(193)	−43.35%	−1.43%	−44.16%
$N_{\text{SCL, polygon}}$ **(SD)**	490(48)	483(108)	865(193)	−43.35%	−1.43%	−44.16%
$N_{\text{V, polygon}}$ [$10^{3}/{\mathrm{mm}}^{3}$] **(SD)**	145.19(14.29)	143.11(32.12)	256.30(57.11)	−43.35%	−1.43%	−44.16%
$N_{\text{V, stereol. EM}}$ [$10^{3}/{\mathrm{mm}}^{3}$] **(SD)**	152.33(11.52)	138.61(29.34)	248.15(54.68)	−	−	−
$N_{\text{V, stereol. seg}}$ [$10^{3}/{\mathrm{mm}}^{3}$] **(SD)**	145.37(9.17)	138.45(31.59)	242.96(58.66)	−	−	−
$N_{\text{V, stereol. EM}}/N_{\text{V, stereol. seg}}$	−4.57%	−0.12%	−2.03%	−	−	−
$N_{\text{V, polygon}}/N_{\text{V, stereol. EM}}$	+4.69%	−3.14%	−3.18%	−	−	−

The number of SCL $N_{\text{SCL}}$ was derived in two different ways, as described in Subsection 2.4. In Table 4, the results for the number of SCL $N_{\text{SCL, graph}}$ and $N_{\text{SCL, polygon}}$ are listed. Both methods delivered identical results. Therefore, it was shown that the number of polygons building the cycle basis of the network is comparable to the number of SCL in the analysed regions of the ACN. Both methods delivered results showing a decrease of the number of SCL $N_{\text{SCL}}$ between the CO and NOX samples of more than 43%. Thus, the connectivity of the ACN was reduced for the NOX sample. A difference between those datasets was qualitatively not identifiable based on SBF‐SEM data and only hardly identifiable based on the 3D ACN reconstructions. Comparing the NOX and HYX animal samples, a slight decrease of $N_{\text{SCL}}$ of 1.43% was detectable, indicating just a slight reduction of the connectivity. Based on SBF‐SEM and 3D ACN reconstruction data, the qualitative differences between those datasets would have been assessed as larger. Comparing the CO and HYX samples, the connectivity was decreased to the largest extent. Summarising, the quantitative ACN analysis revealed differences between the animal samples that would not have been qualitatively observable or would have been incorrectly assessed and estimated by purely qualitative analyses.

The numerical density of SCL $N_{\text{V, polygon}}$ was computed to obtain comparable data to estimations acquired by a manual stereological disector analysis. The numerical density was only computed with the number of polygons $N_{\text{SCL, polygon}}$ contained in the cycle basis of the analysed ACN regions as the results were identical to the results derived from the number of edges and nodes $N_{\text{SCL, graph}}$. $N_{\text{V, polygon}}$ and $N_{\text{SCL, polygon}}$ are linearly correlated, therefore the same percentage deviations between the CO/NOX and NOX/HYX datasets were identified as for the number of polygons $N_{\text{V, polygon}}$.

To assess the ACN segmentation quality, $N_{\text{V, stereol. EM}}$ was compared to $N_{\text{V, stereol. seg.}}$, both obtained by a stereological disector estimation. Results and associated standard deviations for both parameters, as well as the percentage deviations $N_{\text{V, stereol. EM}}/N_{\text{V, stereol. seg.}}$ are listed in the bottom part of Table 4. The deviation in percentage of $N_{\text{V, stereol. EM}}$ to $N_{\text{V, stereol. seg}}$ ranged from 0.12% for the HYX sample, over 2.03% for the CO sample to 4.57% for the NOX sample, with $N_{\text{V, stereol. EM}}$ being higher than $N_{\text{V, stereol. seg}}$ for all datasets.

To test the reliability of the automatically derived quantitative results listed in Table 4, the parameter $N_{\text{V, polygon}}$ was compared to $N_{\text{V, stereol. EM}}$. The percentage deviations for all datasets were determined and are also listed in the bottom part of Table 4. All deviations were less than 5%. For the HYX and CO samples, a slight overestimation of $N_{V}$ of less than 4% by the automated analysis was detectable. For the NOX sample, a slight underestimation of $N_{V}$ of less than 5% by the automated analysis was identified.

## 4 DISCUSSION

In this study, the suitability of a DL approach to generate ACN segmentations on SBF‐SEM data and to perform automated quantitative analyses based on them, was investigated.

The potential of using a DL‐based approach to enhance the segmentation workflow especially regarding the more efficient segmentation of blood cells located within the ACN was highlighted. This approach generated a more complete initial ACN label on test data than the previously established WSM. The qualitative and quantitative analysis of the DL‐based ACN segmentation results showed shortcomings regarding the segmentation of pre‐ or postcapillary blood vessels and false‐positive segmentation results, possibly due to the lack of 3D image context. Moreover, the higher standard deviations for the results of the DL‐based segmentation method compared to the WSM indicated a higher heterogenity of the generated ACN segmentations, which could possibly be improved by considering the 3D context of the SBF‐SEM dataset. Therefore, a 3D DL approach could have the potential to further improve the ACN segmentation results, making the combination with the 3D WSM used in this study unnecessary. Additionally, data augmentation strategies could aid the training process.^54^ However, for this next step, it would be required to include more training and test datasets with corresponding ACN segmentations, which is clearly a restricting condition due to the limited availability of suitable data. At this point, a generalisable fully automated 3D DL‐based ACN segmentation method could not be implemented. Thus, manual interaction during the segmentation process is still necessary. This includes manual completion of the ACN label and removal of excessive ACN segmentation. Nonetheless, the general suitability of using a DL‐based approach to digitally segment the ACN on SBF‐SEM data was shown.

Furthermore, in this study quantitative analyses based on the generated ACN segmentation data were performed. Comparing the quantitative data of the CO and NOX datasets, the number of nodes, edges and SCL were reduced to a similar extent. Thus, it was shown that the ACN connectivity of the NOX sample was reduced compared to the CO sample. Comparing the NOX and HYX datasets, the number of nodes and edges were reduced, but the number of SCL decreased to a much smaller extent. Thus, the ACN connectivity was unsubstantially reduced. The biggest differences occurred between the CO and HYX datasets. The number of nodes, edges and SCL and thus the connectivity were reduced. Based on SBF‐SEM data only the differences between both the CO/NOX and HYX were qualitatively observed. Based on the 3D ACN reconstructions, the differences between the CO and NOX datasets would have been incorrectly assessed in a purely qualitative analysis. Thus, the quantitative analysis revealed ACN differences between the datasets. As the analysis was based on segmentation data, 3D visualisation of the ACN surface was possible without further effort offering context for the quantitative data. Moreover, it was demonstrated that the quality of the ACN segmentation used for the quantitative analysis was sufficient. This was assessed by comparing the numerical density of SCL acquired based on SBF‐SEM data $N_{\text{V, stereol. EM}}$ with the numerical density of SCL acquired based on segmentation data $N_{\text{V, stereol. seg}}$, both obtained by a stereological disector estimation. If the segmentation quality had been low, the deviation of the data would have been large, which was not the case, as all deviations were below 5%. $N_{\text{V, stereol. EM}}$ turned out to be slightly higher than $N_{\text{V, stereol. seg.}}$ which was plausible due to the postprocessing step of removing unconnected parts from the ACN label. The reliability of the automatically obtained quantitative data for the numerical density of SCL was then investigated by comparison of $N_{\text{V, polygon}}$ to $N_{\text{V, stereol. EM}}$, where all deviations were below 5%. For the HYX and CO sample, a slight overestimation of $N_{V}$ by the automated approach was identified, for the NOX sample a slight underestimation. As all deviations were small, the automatically acquired results were assessed as reliable. In this study, it was demonstrated how the acquired quantitative data could be used in a comparative BPD study to investigate the ACN regarding alterations due to prematurity and hyperoxia or prematurity alone if a representative sample size was given like in the light microscopic (LM) study of Rößler et al.^22^ Due to the limited sample size in the present study, comparisons with the previous study are not representative. Only the result for the numerical density of SCL for the NOX sample in the present study was comparable to results acquired by Rößler et al. ($N_{\text{V, stereol. LM}}$ [10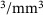] (SD) = 139.48 (18.89)). For the HYX sample, the result was slightly higher than results acquired by Rößler et al. ($N_{\text{V, stereol. LM}}$ [$10^{3}{\text{/mm}}^{3}$] (SD) = 78.36 (22.09)). For the CO sample, the result was almost twice as high as the biggest result obtained by Rößler et al. ($N_{\text{V, stereol. LM}}$ [$10^{3}{\text{/mm}}^{3}$] (SD) = 105.19 (18.89)). As the reliability of the quantitative data was demonstrated and the stereological estimation showed results in the same range, it is likely that these variances occurred due to individual differences between the analysed samples and not due to the applied methods. Another limitation in the current study was the analysis of three subareas per sample. This procedure was chosen as all regions to be analysed were manually checked beforehand to identify areas of low segmentation quality and exclude those from the analysis. Preferably, the entire available datasets would be analysed as a whole connected region to acquire quantitative data delivering an undistorted impression of the ACN architecture of the analysed sample. To achieve this, a sufficient segmentation quality across the entire dataset has to be guaranteed, which is another reason to further improve the segmentation process efficiency. Despite the mentioned limitations, it was shown that an automated quantitative analysis approach based on ACN segmentation data provided reliable information about the ACN. Moreover, it was assessed that the number of polygons contained in the cycle basis of the analysed ACN regions is equal to the number of SCL.

The overall goal for the future is the development of a fully‐automated workflow, beginning at the generation of ACN segmentations of whole SBF‐SEM samples in 3D and ending with an automated quantitative analysis. The software tools for the quantitative analysis are available and deliver reliable results about the ACN architecture, as demonstrated in this study. Furthermore, it was shown that it could be worth taking the DL‐based segmentation approach further. The outcomes of the conducted test using a simple 2D artificial neural network and existing samples, were already encouraging. Given the considerable effort required to implement a DL‐based method, it was essential to initially assess the suitability to process SBF‐SEM data in this way. If the results would have been insufficient at this point, the project would have been cancelled. However, the results presented in this study demonstrated that implementing a DL‐based segmentation approach could prove beneficial. Thus, the next step would be the test of a 3D artificial neural network instead of a 2D method.

While the quantitative analysis approach was demonstrated for the ACN, it could also be used for the analysis of other capillary networks, for example, present in the kidneys or the brain.^55^, ^56^ If segmentation data of sufficient quality is available, a graph representation can be generated, which can be evaluated using the presented automated software tools. At this point, the DL network used in this study is not generalisable due to the limited amount of heterogeneous training data. However, it could be applied for segmentation of different capillary networks, after retraining with appropriate data.

## 5 CONCLUSION

This study showed that DL‐based methods are suitable to segment the ACN on SBF‐SEM data. However, at this point, the efficiency of the segmentation process is still a limiting factor in the overall workflow. It includes manual interaction, like in previous studies,^18^, ^19^, ^20^, ^23^ and combination with other segmentation methods. Compared to these studies, the ACN segmentation efficiency could nevertheless be increased. However, further development of the segmentation approach is required to implement a fully automated ACN analysis method based on segmentation data. With the demonstrated automated software tools, it is at this point possible to obtain reliable quantitative results about the ACN architecture from segmentation data. In contrast to stereological evaluations, this process is completely automated. Thus, for the future it would be desirable to further enhance the described segmentation approach towards an automated generalisable 3D DL‐based segmentation method to enable, for example, quantitative BPD studies or investigations about capillary networks in other organs with representative sample sizes in a reasonable amount of time.

## AUTHOR CONTRIBUTIONS

J.S. and C.M. contributed to the conception and design of the work. J.S., J.L., C.W., Y.R. and J.T. contributed to the acquisition and analysis of the data. J.S., J.L. and C.M. contributed to the interpretation of the data. All authors reviewed and approved the submitted manuscript.

## CONFLICT OF INTEREST STATEMENT

The authors declare no potential conflict of interests.

## CODE AVAILABILITY

Makefiles used to manage the workflow steps are available upon request.

## Data Availability

The datasets generated and analysed during the current study are available on request.
